# The Sensor-Based Biomechanical Risk Assessment at the Base of the Need for Revising of Standards for Human Ergonomics

**DOI:** 10.3390/s20205750

**Published:** 2020-10-10

**Authors:** Alberto Ranavolo, Arash Ajoudani, Andrea Cherubini, Matteo Bianchi, Lars Fritzsche, Sergio Iavicoli, Massimo Sartori, Alessio Silvetti, Bram Vanderborght, Tiwana Varrecchia, Francesco Draicchio

**Affiliations:** 1 Department of Occupational and Environmental Medicine, Epidemiology and Hygiene, INAIL, Monte Porzio Catone, 00040 Rome, Italy; s.iavicoli@inail.it (S.I.); al.silvetti@inail.it (A.S.); t.varrecchia@inail.it (T.V.); f.draicchio@inail.it (F.D.); 2 HRI2 Laboratory, Istituto Italiano di Tecnologia, 16163 Genova, Italy; arash.ajoudani@iit.it; 3 LIRMM, University Montpellier, CNRS, 34000 Montpellier, France; andrea.cherubini@lirmm.fr; 4 Centro di Ricerca “Enrico Piaggio” and Department of Information Engineering, Università di Pisa, 56126 Pisa, Italy; matteo.bianchi@centropiaggio.unipi.it; 5 Ergonomics Division, IMK Automotive GmbH, 09128 Chemnitz, Germany; lars.fritzsche@imk-automotive.de; 6 Department of Biomechanical Engineering, University of Twente, 7522 NB Enschede, The Netherlands; m.sartori@utwente.nl; 7 Brubotics, Vrije Universiteit Brussel, 1050 Brussels, Belgium; bram.vanderborght@vub.ac.be; 8 Flanders Make, Oude Diestersebaan 133, 3920 Lommel, Belgium

**Keywords:** wearable sensors, sensor-based biomechanical risk assessment, International Standards for ergonomics, human–robot collaboration technologies

## Abstract

Due to the epochal changes introduced by “Industry 4.0”, it is getting harder to apply the varying approaches for biomechanical risk assessment of manual handling tasks used to prevent work-related musculoskeletal disorders (WMDs) considered within the International Standards for ergonomics. In fact, the innovative human–robot collaboration (HRC) systems are widening the number of work motor tasks that cannot be assessed. On the other hand, new sensor-based tools for biomechanical risk assessment could be used for both quantitative “direct instrumental evaluations” and “rating of standard methods”, allowing certain improvements over traditional methods. In this light, this Letter aims at detecting the need for revising the standards for human ergonomics and biomechanical risk assessment by analyzing the WMDs prevalence and incidence; additionally, the strengths and weaknesses of traditional methods listed within the International Standards for manual handling activities and the next challenges needed for their revision are considered. As a representative example, the discussion is referred to the lifting of heavy loads where the revision should include the use of sensor-based tools for biomechanical risk assessment during lifting performed with the use of exoskeletons, by more than one person (team lifting) and when the traditional methods cannot be applied. The wearability of sensing and feedback sensors in addition to human augmentation technologies allows for increasing workers’ awareness about possible risks and enhance the effectiveness and safety during the execution of in many manual handling activities.

## 1. Introduction

This Letter intends to address a crucial issue of the occupational field concerning the prevention of work-related musculoskeletal disorders (WMDs). In particular, the authors of this Letter aim to analyze the need for a revision of ergonomics standards in order to include the use of new sensor-based approaches for the monitoring of workers’ motor activities and biomechanical risk assessment. The main reasons why this review is considered necessary are discussed in more detail below:

- Some implicit limitations of several traditional methods listed within the international ergonomics standards developed in an attempt to prevent and reduce the risk of WMDs, able to identify manual handling activities associated with a high risk of WMDs and to evaluate the effectiveness of ergonomic interventions [1,2];
- The new opportunities represented by innovative wearable devices for workers monitoring and feedback;
- The new “Industry 4.0” scenario which is making these methods increasingly difficult to apply. Indeed, the presence of new human augmentation technologies in many manual handling activities is not currently included in the standards with the consequent difficulty of associating a biomechanical risk with these tasks;
- The criticisms, underlined by the literature, related to scientific basis these international standards were created on.

The European Union (EU) is contributing significantly to the huge international effort in this field by recognizing collaborative robotics as one of the technologies that can positively affect the economy and society. To that end, the EU has defined the Strategic Research Agenda and the multi-annual Roadmap, which provides a strategic overview and technical guide identifying medium term research and innovation goals [3,4] for the robotic community. Furthermore, the EU encourages standardisation activities in EU projects for a better market adoption and to develop a single digital market [5].

In particular, the European Union’s Horizon 2020 Research and Innovation Program funded, under grant agreement No. 871237, the project “SOPHIA—Socio-Physical Interaction Skills for Cooperative Human–Robot Systems in Agile Production” [6]. SOPHIA aims at developing a new generation of human–robot collaboration (HRC) technologies (namely, human augmentation technologies such as wearbots and cobots) able to improve, among others, human ergonomics during the execution of occupational manual handling activities. Wearbots, such as exoskeletons, are defined as wearable under-actuated devices with advanced interaction and sensing capabilities, designed to offload workers from internal loadings and to keep them in ergonomic and comfortable working conditions [7,8]. Cobots are defined as reconfigurable collaborative robots, capable of responding to task variations and to the worker’s intentions in a timely manner, while simultaneously offloading him/her from external loadings (task-related payloads) and keeping him/her in task-optimum and ergonomic working conditions. These HRC technologies will embed new instrumental-based tools for anticipating and evaluating human physical states during work [9]. Technological advances deriving from the project activities will allow a better management of the challenging occupational health problems represented in Europe by WMDs which imply sick leave, disability, and early work interruption [10,11].

To ensure the underlying design ‘compliance’ to standards, the project will focus on the revisions of already existing European and/or international standards and/or formulating a proposal for the development of novel Standards in the field of human ergonomics and HRC. In particular, standardization activities for ergonomics are necessary, since the epochal changes due to “Industry 4.0” [12] era are widening the number of work tasks that cannot be assessed with traditional international standards. New standards—their proper dissemination and translation into good practices—will create conditions for economic growth. In fact, new standards will enable an easier transfer of research results to the market, have a significant socio-economic impact and allow a better agreement on common specifications and procedures that respond to the needs of business and meet consumer expectations.

Standardization activities in the field of ergonomics should be planned as follows:

- Overview of existing standards in the field of biomechanical risk assessment, to identify gaps in the standardization repository;
- Recommendations for modifying existing standards in the field of biomechanical risk assessment, by analyzing strengths and weaknesses of various methods listed within the standards, and by considering the new HRC scenario;
- Identification of standardization potential and needs, aimed at developing new standards or to create a roadmap of new standardization activities.

As a first step, this Letter aims at highlighting the needs for revising standards for human ergonomics and biomechanical risk assessment presenting some insights that may be useful to start a discussion. The idea is to consider the limitations of the previously published standards during the future revisions of the international standards considering the possibility of including the use of sensor-based tools for the assessment of WMDs. Of course, the reliability and validity of the use of these sensors warrants further investigations. This aim was achieved by:

- Underlining the huge problem linked to the onset of WMDs reporting a synthesis of their current incidence and prevalence in several world countries (Section 2);
- Assessing strengths and weaknesses of methods and international standards for manual handling activities, especially in view of the new technological opportunities (wearable sensors for monitoring and HRC systems) offered by industry 4.0 (Section 3);
- Discussing contents and next challenges needed for the revision of the international standards (Section 4).

## 2. WMDs: Definitions, Prevalence and Incidence

WMDs are a set of painful inflammatory and degenerative conditions, affecting the joints, spinal discs, cartilage, muscles, tendons, ligaments and peripheral nerves. The work environment and performance of work contribute considerably to the WMDs conditions which is worsened or prolonged due to unfavorable work circumstances [13].

WMDs are associated with specific physical risk factors of manual handling including manual heavy lifting, repetitive arm and hand movements such as handling low loads at high frequency and computer work, and awkward body postures [14]. These work activities can determine loads at L5-S1 spine joint exceeding the tissue tolerance [15,16,17], increased trunk antagonist muscle co-activations [18,19,20,21,22,23] and local muscle fatigue [24,25,26,27,28,29,30,31].

Although an accurate analysis on WMDs incidence and prevalence is difficult to perform and to compare across countries, below we present a summary of studies conducted in different countries around the world. In particular we found studies from Australia, Europe (Portugal, France, The Netherlands, Great Britain and Italy), America (USA and Canada) and Asia (Korea). The 7-day prevalence of WMDs is 42%, of which 19% with symptoms in the lower back, 17% in the wrist and hand, 16% in the neck, 15% in the shoulder, 25% in feet, ankles, knees and hips, 5% in the elbows and 3% in the dorsal region [32]. Among WMDs, work-related low-back disorders (WLBDs) and upper limb work-related musculoskeletal disorders (UL-WMSDs) have a 12-month worldwide prevalence ranging from 12% to 41% [33,34,35]. These data show that the upper body district is the most involved, although recently the lower extremities have received more attention. The annual incidence of WMDs ranges between a quarter and a third of all occupational diseases [1,36,37,38,39,40,41,42]. Findings of the above cited studies were obtained mainly using interviews and validated questionnaires but also video recordings and/or physical examinations.

## 3. International Standards for Manual Handling Activities: Strengths and Weaknesses

In the last decades, several international standards have been developed to prevent WMDs. The International Standard Organization (ISO) 11228 parts 1, 2 and 3, 11226, the technical reports 12295 and 12296 [43,44,45,46,47,48] have the dual objective of allowing the detection and rating of the risk condition levels and assessing the efficacy of appropriate ergonomic interventions on the reduction of biomechanical risk. The above-mentioned international standards, referring to all the manual handling activities that determine the onset of WMDs, list several methods that require measuring specific parameters (i.e., forces, frequencies, joint angles, etc.) which in turn define multipliers and/or addends used in equations, to provide risk level scores. Table 1 reports an extensive list of these methods [2,49,50,51,52,53,54,55,56,57,58,59,60,61,62,63,64,65,66,67,68,69,70,71,72,73,74,75,76,77,78,79,80,81,82,83,84,85,86,87,88,89,90,91,92,93,94,95,96] used for the biomechanical risk assessment.

The main strength of these approaches is their low-cost and non-invasive nature. However, they are associated with some weaknesses [97] such as:

- Their observational nature has limited the scalability and generality of the standards;
- They produce results that usually include some subjectivity;
- They are usually pen-and-paper based, hence time consuming and inefficient;
- They are influenced by the restrictions of the equations and parameters;
- They may not have sufficient accuracy, precision and resolution;
- They may not be repeatable and reliable, which is indispensable for industrial use.

Other than the above listed issues widely debated in the literature, the standardization activities discussed will consider and contribute to the indispensable debate promoted by Armstrong and colleagues, within an interesting discussion paper [98] analyzing the scientific basis of ISO standards on biomechanical risk factors. The discussion has already been enriched by two other contributions [99,100], focused on the scientific basis of the occupational repetitive action (OCRA) method for risk assessment of biomechanical overload of upper limb.

The authors of the discussion paper made several criticisms related to the ISO 11226 and ISO 11228 series that we summarize as follows:

- Need for procedures for producing ISO ergonomics standards other than writing evidence-based practical guidelines (e.g., no presentation of the methods used for selecting the recommended force limits and risk assessment tools, no adoption of transparent scientific review processes);
- Absence of information about criteria for identification of subcommittees: the identities of subcommittee members are undisclosed and their scientific profiles are not described;
- Undefined involvement of the key stakeholders: labour authorities, companies, ergonomics professionals and knowledgeable scientists are crucial for ISO actions and for improving the knowledge and expertise. They are needed to apply the risk assessment methods appropriately and professionally at workplace. Properly chosen stakeholders should also provide external peer-review;
- Unclear choices of the preferred methods of risk assessment over others: this issue is crucial also to avoid potential conflict of interests;
- Statements based on personal opinions and in contrast with the literature.

The above-mentioned issues alone may be sufficient for a rigorous update of the methodologies and results. In addition to that, we recommend that other important aspects, leading in the same direction of a significant renovation in this field, should be taken into consideration. In particular, new technological advances, regarding wearable sensors for biomechanical risk evaluation and adaptable workplaces, are extensively changing both movement monitoring and worker task performances.

The new scenarios contrast with the fact that the procedure used at least a decade ago led to methods, currently accepted within the ISO 11228 series, which cannot take into consideration these technologies. The most recent innovative miniaturized wireless wearable sensors attached to the workers body (e.g., inertial measurement units (IMUs), insoles for measuring reaction forces and surface electromyography (sEMG) sensors, etc.), as well as 3D depth cameras measuring his/her motion can strongly enhance accuracy and precision of the biomechanical evaluation via data-model fusion techniques [21,22,101,102,103,104]. These devices could be used for both quantitative “direct instrumental evaluations” and “rating of standard methods”, allowing certain improvements over traditional approaches [97]. Direct instrumental evaluations could be used for sensor-based biomechanical risk assessments when existing methods are not usable or, when usable, to obtain confirmation of their goodness. The rating of standard methods could be used to measure some parameters, otherwise measured with poor precision and accuracy, necessary to obtain the level of risk. For example, many traditional methods for assessing the biomechanical risk of handling low loads at high frequency activities require the measurement of the upper limb joint range of motions, which can be easily and automatically calculated by using IMUs and dedicated algorithms.

Furthermore, the significant changes at the workplace (in both large industries and small-medium enterprises) and the increasing use of human–robot collaboration technologies (remotely controlled robots, occupational collaborative robots and wearable exoskeletons) [9,105,106,107,108,109,110,111,112,113] including sensory feedback devices [102,114] have all shown their potential for reducing biomechanical effort and work-related injuries. The current methods for biomechanical risk assessment do not take into consideration these assistive technologies, which open yet another opportunity for revising the existing standards for human ergonomics.

## 4. Discussion

The four-years SOPHIA project research agenda has identified the prevention of WMDs as one of its key objectives, by focusing on the multifactorial relationship between manual handling activities and musculoskeletal health [115]. The other objectives can be summarized in the creation of a new generation of HRC technologies able to increase the flexibility and productivity of manufacturing while improving the ergonomics of the workplace. These systems will be inexpensive, accurate, precise, of low complexity and will provide a quantitative assessment. To this end, the project envisions a large and ambitious research and innovation program to focus on core technology, usability, acceptability and standardization objectives. In particular, it will be necessary to take into account, within the methods for biomechanical risk assessment, work task features and individual biomechanical and physiological factors, which can be considered the main determinants of WMDs [116]. With this aim, the SOPHIA project planned research activities with the goal of analyzing the scientific foundations on which the revised/new standards will be based on, by considering both traditional and instrumental-based biomechanical assessment tools, to take into account the new generation of working tasks and workplace settings.

The strategy for obtaining an effective revision of the ergonomics standards is strongly linked to the need to have international standards that follow, to the extent possible, an evidence-based scientific approach consisting of the following actions:

- For each kind of work task (lifting, handling low loads at high frequency, overhead work, etc.) a systematic search and appraisal of the available findings published in international peer-reviewed journals should be performed on the sensor-based biomechanical risk assessment topic;
- The ISO Standards should consider only the approaches evaluated in large prospective and cross-sectional studies;
- The ISO Standards should provide any information on the reduction of the risk of WMDs expected for any given level of exposure;
- The ISO Standards should consider an external peer-review by key stakeholders, relevant professional societies and interested scientists.

As we have seen in Table 1, the international standards report many methods on which it is impossible here for everyone to make an in-depth analysis. As a representative example, in this discussion section we focus only on lifting heavy loads. We do this because the mean percentage of time spent by European workers in carrying or moving heavy loads is 32%, and it varies within a range of 24–41% [117]. A pioneering tool implemented by the ISO 11228–1 and commonly used for prevention of WLBDs is based on the revised NIOSH (National Institute for Occupational Safety and Health) lifting equation (RNLE), which provides an evaluation of the physical effort associated with lifting activities [2,118]:

(1) $$m_{R}=m_{ref}xh_{M}xv_{M}xd_{M}x\alpha_{M}xf_{M}xc_{M}$$

where *m_R_* is the recommended weight limit, *m_ref_* is the reference mass for the identified user population group, *h_M_*, *v_M_*, *d_M_* and *α_M_* are multipliers calculated respectively starting from the horizontal distance, vertical location, vertical travel displacement of load and angle of asymmetry, $c_{M}$ is the coupling multiplier for the quality of gripping and $f_{M}$ is the frequency multiplier depending on lifting frequency, lifting duration and vertical location. The lifting index (LI) is calculated as the ratio between the actual weight lifted ($m_{A}$) and the recommended weight limit m_R_. The risk of WLBDs has been shown to increase as the LI increases from 1.0 to 3.0, with a significant odds ratio [119,120,121,122].

Among ergonomic interventions, a good work organization, the willingness of workers to wear exoskeletons [123] and the use of appropriate aids can significantly contribute in reducing biomechanical risk during the execution of the lifting activity [43]. In some work conditions, collaborative lifting (team lifting) executed by more than one person is a common practice, and it represents a good safety rule for reducing musculoskeletal disorders. The team lifting is necessary when the loads to be lifted overcome the threshold limits proposed for safe lifting by one worker [118,124] and when a mechanical transportation, which could be used with high loads, is either unavailable or impractical [125]. To consider team lifting with two or three people, the RNLE has been further extended in TR 12295 [46]. Despite this effort, the TR 12295, as well as the other technical reports, includes mainly informative contents, which are very different from those provided by the ISO 11228, since it includes data obtained from surveys, informative reports or information of the perceived “state of the art”.

For instance, again concerning team lifting, the TR 12295 suggests a development of the RNLE derived from the ISO 11228–1 to consider lifting performed by two or three people modifying the RNLE with a further multiplier $p_{M}$ that is the persons multipliers whose value is 0.67 and 0.5 for two and three persons lifting activities respectively. The corresponding LI equations for 2 or 3 persons ($LI_{2pt},\;LI_{3pt}$) are calculated as the ratio between the $m_{A}$ and the m_R_ multiplied by 1/2 ×  2/3 and 1/3 × 1/2 respectively.

This model shows two main issues. By a mathematical point of view the LI equation should be modified. By the motor strategy point of view, it would be much more appropriate to measure the team lifting LI by using wearable sensors and appropriate biomechanical calculations instead of an “approximate guide”. In fact, the standard should take into account that a reduction of both the spine loading and the biomechanical risk occurs only if the team lifting is performed correctly. Indeed, the two or three people lifting requires much greater coordination, precision, synchronization and control between lifters than a one-person lifting. The team has to coordinate precisely, while it would be possible for a one-person lift to place simply and smoothly the load at the destination point [126]. Furthermore, the problem of irregular loading in a team may be even more compounded if team members are not comparable in factors such as strength, height, lifting experience, and perception. The way load and effort are distributed among team members could be influenced also by other factors like size and/or shape of the load the load, task and environment characteristics [127]. The apparent decrement in team lifting compared to individual lifting performance expressed as a proportion of summed individual capabilities, may be partially explained by “social loafing”, a term that describes reduced effort by individuals when they work in a team [128]. Along with losing autonomy and coordination, lifting teams may also find it difficult to assess their total team-lifting capacity [127].

The above-mentioned factors influencing the team capacity to lift heavy loads suggest that the best—and probably most unique—way of evaluating the team lifting is to use kinematic, kinetic and sEMG instrumental-based approaches [20,25,97,129,130]. Our conviction is also supported by the possibility of being able to use computational biomechanical modeling or machine-learning algorithms such as artificial neural networks to optimise risk classification [131,132]. Finally, instrumental-based tools may also rate the risk for hybrid lifting tasks, performed collaboratively by workers and robots. Typically, in the context of HRC, present day cobots all embed force and kinematic sensing in their joints, making a direct and objective evaluation metric readily and always available during the collaborative task. On the other hand, guaranteeing the aforementioned team lifting requirements (coordination, precision and synchronization) with a cobot is still challenging nowadays.

Further findings of the last decade—focused on RNLE—suggest additional changes regarding the values of both $m_{R}$ [133] and LI [134]. The first draft of the ongoing ISO 11228–1 revision, while introducing changes such as the composite lifting index (CLI) [2], (i.e., lifting a similar weight with different geometries) and the variable lifting index (VLI) [135] (i.e., handling different weights with variable geometries), does not take into consideration the aspects mentioned in this paper.

Therefore, the most relevant challenges with respect to RNLE are:

- The use of instrumental-based tools for rating the RNLE parameters;
- The design of new RNLE multipliers, capable of rating the risk during lifting tasks performed by HRC technologies, such as cobots and wearbots. For instance, in recent years, new wearable assistive devices such as exoskeletons have been introduced in the workplace and their use is expected to become more commonplace. In particular, exoskeletons appear to be a new option in addressing WMDs [136]. An advantage of both wearbots and cobots is that both are generally sensorized, a feature which facilitates risk evaluation. The wearability of sensing and feedback devices (fellow–feeling wearables) in addition to fellow–assistant wearbots and cobots allows for increasing workers’ awareness about possible risks and enhance the effectiveness and safety during the interaction with cobots and robots.
- The design of new instrumental-based approaches are capable of directly quantifying the biomechanical risk, when the RNLE cannot be applied. For instance, the execution of hybrid team lifting tasks implies complex coordination mechanisms that need to be studied [125].

These and similar challenges should be at the base of the next revisions needed for existing standards regarding other manual handling activities, such as handling low loads at high frequency.

A potential development of the use wearable sensor-based biomechanical risk assessment methods is its future integration with data-driven computational biomechanics [104,137,138]. This is a data-model fusion approach that will enable observing a range of neuromuscular variables that is larger than what could be observable using sensors or computational models alone (i.e., muscle activation, muscle/tendon force and joint stiffness and damping) [139,140]. In this context, a pure sensor-based approach would not enable sampling internal neuromuscular variables (i.e., individual muscle force or stiffness) in vivo in the intact moving human in a non-invasive way. On the other hand, the ability of using non-invasive wearable sensor data (e.g., electromyography surface electrodes, thin-film low-profile ultrasonography probes) to drive forward subject-specific neuromuscular models will lead to a framework for deriving high-fidelity estimates of human neuromuscular function [141,142]. This is expected to provide new avenues to inform wearable device controllers of the user’s current physiological state or determine the optimal body postures to prevent musculoskeletal injuries on the long term [7,8,143].

## 5. Conclusions

In conclusion, the authors of this letter believe that a biomechanical risk assessment of manual handling tasks based on wearable sensors for monitoring and feedback can be a key approach leading a revision of International Standards for human ergonomics. This revision process is mainly necessary because the use of innovative human augmentation technologies in the workplace makes traditional methods not applicable. Fortunately, also these new technologies such as wearbots and cobots embed miniaturized sensors that make the quantitative instrumental-based biomechanical risk assessment even easier.

## Figures and Tables

**Table 1:** Methods used for biomechanical risk assessment.

Method	Work Activity	Body Part Assessment	References
Revised NIOSH lifting equation (RNLE)	Lifting and carrying	Shoulders, trunk, hands	[2]
Key indicator method (KIM-MHO)	Lifting and carrying	Trunk, hands	[49,50]
Manual handling assessment chart (the MAC tool)	Lifting and carrying	Trunk	[51]
LBP as a function of patient lifting frequency	Patient handling	Trunk	[52]
Back injury prevention project (BIPP)	Patient handling	Trunk	[53]
PATE	Patient handling	Trunk	[54]
DINO	Patient handling	Trunk	[55]
Patient handling assessment	Patient handling	-	[56]
Patient transfer assessing instrument (PTAI)	Patient handling	Whole body	[57]
MAPO	Patient handling	-	[58,59]
TilThermometer	Patient handling	-	[60]
Manual handling assessments in hospitals and the community	Patient handling	-	[61]
Revised tables of maximum acceptable weights and forces	Pushing and pulling	-	[62]
Mital Tables	Pushing and pulling	-	[63]
Risk assessment of pushing and pulling tool (RAPP)	Pushing and pulling	Trunk	[64]
Assessment of pulling and pushing based on key indicators	Pushing and pulling	Trunk	[65]
RAMP tool	Pushing and pulling	Trunk	[66]
ACGIH assessment of hand activity level (HAL)	Repetitive movements	-	[67,68,69,70]
Strain index (SI)	Repetitive movements	Hands, wrists	[71,72]
Occupational repetitive actions (OCRA) index and checklist	Repetitive movements	Shoulders, elbows, wrists, hands	[73,74,75,76,77,78,79]
Quick exposure check (QEC)	Repetitive movements	Upper limbs, back	[80,81,82,83]
Outil de repérage et d’evaluation des gestes (OREGE)	Repetitive movements	Upper limbs	[84]
OWAS	Repetitive movements	Whole body	[85]
Posture, activity, tools and handling (PATH)	Repetitive movements	Whole body	[86]
Rapid upper limb assessment method (RULA)	Repetitive movements	Whole bodyParticular attention to the neck, trunk, shoulders, arms, wrists	[87,88]
Rapid entire body assessment method (REBA)	-	Trunk, legs, neck, shoulders, arms, wrists	[89,90]
The European Assembly Worksheet (EAWS)	Repetitive tasks	Upper limbs	[91]
Assessment of repetitive task (ART)	Repetitive tasks	Upper limbs, neck, trunk	[92]
Upper limb risk assessment (ULRA)	Repetitive tasks	Arms, forearms, hands	[93]
Manual Handling Assessment Chart (MAC)	Manual material handling tasks	Shoulders, Trunk	[94,95]
Agricultural lower limb assessment (ALLA)	Agricultural tasks	Lower limb	[96]

- indicates “not specified”.
